# Important Aphorisms. Applied to Plate Work

**Published:** 1910-09-15

**Authors:** L. P. Haskell

**Affiliations:** Chicago


### IMPORTANT APHORISMS. APPLIED TO PLATE WORK.

BY L. P. HASKELL, CHICAGO.

Don't forget:

1. That plaster is always a reliable impression material.

2. That the more difficult the case to obtain an impression, the more necessary the plaster.

3. That the only portion of the upper jaw which never changes is the hard palate.

4. That unless provision is made for the settling of the alveolar ridge, it is only a question ot time when the plate is resting and rocking on the palatel surface.

5. That the vacuum cavity is not at all needed to retain the plate, and, also, if used is sooner or later rocking the plate.

6. That the remedy, in a metal plate, is the covering of the entire hard surface with a thin film of wax (the "relief"). In a vulcanite plate, scraping the impression.

7. That there is no necessity nor advantage in scraping the soft portions of the model in any case.

8. That vulcanite should not be used for permanent upper dentures, because of increased absorption in 80 per cent. of mouths on account of retention of undue heat.

9. That the great number of cases of excessive absorption of the anterior portion of the upper jaw and soft ridge arises from this cause, but is greatly enhanced by undue pressure of the anterior teeth.

10. That the anterior teeth in full upper dentures should *never ,never,* under any conditions, come in contact for the above reason, and also because the plate is displaced every t me the jaws meet.

11. That the filling of a metal plate is as easy as of a vulcanite, and in flat, ridgeless jaws better success is assured.

12. That a proper Babbit metal die insures better success than zinc, because it has all of the five necessary qualities for a dental die; viz., non-shrinkage, will not batter, will not break, is smooth and melts at a low temperature.

13. That the melting temperature of the lead for counter-die must be reduced by the addition of tin, one part, to five of lead, and not poured as hot as it comes from the heater, but stirred until it becomes to crystallize.

14. That aluminum makes an excellent substitute for vulcanite.

15. That in the question of the extraction of certain teeth, the only thing to be considered is what shall be done to make the artificial denture the most useful and comfortable.

16. That the retention of the cuspid teeth is unwise from every point of view, weakening the denture; the latter is not so easily retained, nor as useful.

17. That there is no necessity for retaining them, because they are practically useless, and the change in the features caused by their extraction is remedied by making the plate higher at those points and the artificial gum fullest.

18. That there are more failures from faulty occlusion than from any other cause.

19. That direct occlusion can better be secured by the use of the thick articulation paper than by any other means.

20. That in arranging the lower to an upper set, begin with the second bicuspids; then the first, so as to secure correct interlocking of the cusps ,for in nearly all full sets, of all makes, the anterior lower teeth are too wide for the uppers, and must be changed so as to come within their proper limits.

21. That in taking the bite“”, if the tongue is turned back as far as possible the jaw cannot be moved forward.

22. That teeth should always be arranged by the mouth, as it is only there one can determine when they are correct, and also the patient should see them, so if any change is desired it can be made prior to completion.

23. That the numerous cases of flat, narrow, ridgeless lower jaws are the problem of the dentist.

24. That when the tongue is raised the glands and loose integuments rise above and drop over the margin of the jaw.

25. That in such cases, no matter what depth there is on the lingual side of the jaw, the plate should not be extended below the point where it is lifted by the glands, as it is constantly lifted, to the great annoyance of the wearer.

26. That a very common fault with artificial rteeth in many mouths is they are too short, no attempt being made to restore the features.

27. That small, white or colorless teeth are too often used, and resemble a row of beans.

28. That the serious fault with all makes of teeth is found in the bicuspids and molars. The lingual cusps should be shorter than the buccal in the upper teeth, and as they are not, it is impossible to bring the buccal cusp into proper alignment without much grinding of the lingual.

29. That the pins, even in long teeth, are too near the cusp when it has to be ground nearly all the way, whereas the pins should be lowered, allowing more porcelain and, cusp when it has to be ground nearly all the way, whereas the pins should be lowered, allowing more porcelain and, also, shorter cusps.

30 .That too many bicuspids and molars are so narrow and thin there is little surface for mastication.

31. That the continuous gum denture remains, after nearly fifty-eight years, the only ideal artificial denture in all respects.

32. That the prominent upper jaw and short lip absolutely requires this work to fulfill all the requirements of the case.

33. That nothing else in prosthesis gives such scope for artistic work, and yet much of it is a disgrace to the maker.

34. That there are seven distinct peculiarities of the left side of the mouth, seldom seen on the right, all of which have to do with the arrangement of the teeth and contouring of the gums.—*Dental Digest.*

